# *Plasmodium* curtails autoimmune nephritis via lasting bone marrow alterations, independent of hemozoin accumulation

**DOI:** 10.3389/fimmu.2023.1192819

**Published:** 2023-07-19

**Authors:** Laura Amo, Hemanta K. Kole, Bethany Scott, Chen-Feng Qi, Ludmila Krymskaya, Hongsheng Wang, Louis H. Miller, Chris J. Janse, Silvia Bolland

**Affiliations:** ^1^ Laboratory of Immunogenetics, Division of Intramural Research, National Institute of Allergy and Infectious Diseases, National Institutes of Health, Rockville, MD, United States; ^2^ Laboratory of Malaria and Vector Research, National Institute of Allergy and Infectious Diseases, National Institutes of Health, Rockville, MD, United States; ^3^ Leiden Malaria Research Group, Department of Parasitology, Leiden University Medical Center, Leiden, Netherlands

**Keywords:** *Plasmodium*, lupus, nephritis, autoimmune, FcgammaRIIB, SLE

## Abstract

The host response against infection with *Plasmodium* commonly raises self-reactivity as a side effect, and antibody deposition in kidney has been cited as a possible cause of kidney injury during severe malaria. In contrast, animal models show that infection with the parasite confers long-term protection from lethal lupus nephritis initiated by autoantibody deposition in kidney. We have limited knowledge of the factors that make parasite infection more likely to induce kidney damage in humans, or the mechanisms underlying protection from autoimmune nephritis in animal models. Our experiments with the autoimmune-prone FcγR2B[KO] mice have shown that a prior infection with *P. yoelii* 17XNL protects from end-stage nephritis for a year, even when overall autoreactivity and systemic inflammation are maintained at high levels. In this report we evaluate post-infection alterations, such as hemozoin accumulation and compensatory changes in immune cells, and their potential role in the kidney-specific protective effect by *Plasmodium*. We ruled out the role of pigment accumulation with the use of a hemozoin-restricted *P. berghei* ANKA parasite, which induced a self-resolved infection that protected from autoimmune nephritis with the same mechanism as parasitic infections that accumulated normal levels of hemozoin. In contrast, adoptive transfer experiments revealed that bone marrow cells were altered by the infection and could transmit the kidney protective effect to a new host. While changes in the frequency of bone marrow cell populations after infection were variable and unique to a particular parasite strain, we detected a sustained bias in cytokine/chemokine expression that suggested lower fibrotic potential and higher Th1 bias likely affecting multiple cell populations. Sustained changes in bone marrow cell activation profile could have repercussions in immune responses long after the infection was cleared.

## Introduction

Infection with *Plasmodium* causes a strong activation of innate immune responses and inflammatory cytokines in the host, due to the sensing of nuclei-acids associated with replicating parasite, parasite-derived factors and red blood cell (RBC) damage (1–4). Subsequently, infected RBCs are phagocytized by myeloid cells and can initiate adaptive immune responses (5). When the initial response against the parasite subsides, anti-inflammatory and regulatory mediators, such as TGF-β, IL-10 and Tregs, temper the immune response so as not to overwhelm the host (6, 7). Initial exposure often causes clinical disease, but subsequent repeated exposures result in partially protective, non-sterile immunity. This, together with the finding of detectable signs of alteration in their circulating monocyte sub-populations, suggests that repeated malarial infections may affect the development of effective immune responses in future challenges (1, 8–11). In addition, various studies on human malaria and murine models have revealed an impaired dendritic cell (DC) function and maturation after infection (12, 13), although the details of this effect are still unclear.

Our own work on the effect of *Plasmodium* on the progression of autoimmune disease has pointed to a long-lasting modification of DC biology that impacts severe kidney disease while sparing other autoimmune manifestations (14). These studies were based on the observation that malaria can have a suppressive effect on rheumatic diseases, a hypothesis first voiced decades ago by Greenwood et al. (15, 16). The interconnection between malaria and autoimmune disease (SLE primarily) provides a prime example of the enduring effect of malaria in chronic inflammatory conditions. This effect has been reported with correlating evidence in human populations (17–22) and mechanistic insights in various murine models (23–25). Autoantibodies are frequently reported in cases of human malaria. Acute renal failure is a serious complication of severe malaria with *P. falciparum* but it rarely progresses to lethal end-stage kidney disease. Immune complex deposition in the kidney has been proposed as one cause of renal failure in human malaria (26).

Among the studies in autoimmune models, the first report came from the group of Brian Greenwood, who revealed a protective effect of murine *Plasmodium* from lethal nephritis in the lupus mouse model NZB/NBW (15). Our group has more recently reported similar findings with the autoimmune-prone FcγR2B-deficient (R2) mice (14). R2 mice develop spontaneous chronic disease characterized by the presence of circulating autoantibodies and a systemic inflammatory response that ultimately causes lethal nephritis via infiltration of myeloid cells and T cells into the kidney (27). Prior infection with *P. yoelii* 17XNL (*Py*) at young age (before the appearance of autoimmunity) improved survival rates of R2 mice due to reduced infiltration of bone marrow (BM)-derived leukocytes into the kidney (14).

Thus, it is of interest to uncover mechanisms by which *Plasmodium* modifies BM populations in a manner that can impact autoimmune nephritis. In particular in the current study, we have considered two plausible processes that could explain how *Plasmodium* alters BM cells (1): the presence of persisting *Plasmodium*-derived products such as the pigment hemozoin (Hz) might alter phagocytic cell biology and (2) the strong inflammatory reaction against the parasite might bias immune cells into a specific type of maturation state that precludes later outcomes (i.e. severe nephritis).

*Plasmodium*-derived Hz crystals accumulate in various organs as a byproduct of erythrophagocytosis and might have an immunomodulatory effect (28–30). It has been reported that Hz impairs DC maturation and function (31–40), reduces macrophage inflammatory responses and monocyte migratory profile as well as inhibits erythroid development (41–43). Work presented here demonstrates that the protective effect on lethal glomerulonephritis due to *Plasmodium* does not correlate with the extent of Hz production. For this work we use various *Plasmodium* lines, one of which produces little Hz while still conferring protective effect on autoimmune nephritis (29, 44, 45). We observe that, regardless of strain, *Plasmodium* infection alters the BM environment and biases cytokine production away from the type that are responsible for fibrotic damage in autoimmune nephritis. Thus, these results provide an additional example of the long-term effect of malaria infection on the quality of subsequent immune responses.

## Materials and methods

### Experimental animals

FcγRIIb[KO] (R2, Taconic TAC264) mice were maintained in specific pathogen free SPF conditions at the National Institutes of Health. Congenic CD45.1^+^ R2 mice were generated by crossbreeding TAC264 mice with JAX strain 002014. C57Bl/6J mice were obtained from the Jackson Laboratories. All animals were housed and studied under the approved NIH ACUC guidelines and the experiments were carried out in accordance with the approved NIH Animal Study Protocol guidelines. All efforts were made to minimize animal suffering and to reduce the number of animals used.

### *Plasmodium* strains and infection

The rodent malaria parasites used in this work are *Plasmodium yoelii* 17XNL GFP (*Py*) (46) and two *Plasmodium berghei* ANKA (*Pb*) mutants. The mutant Δpm4-Δbp2/GFP-Luc@ama-1Δpm4-Δbp2 (*Pb* Δpm4-Δbp2), which lack expression of the enzymes plasmepsin 4 (PM4) and berghepain-2 (BP2). Blood stages show a strongly reduced hemoglobin digestion and Hz formation (44), and parasites can grow and multiply in reticulocytes without the formation of Hz (29, 45). In addition, mutant Δlap/GFP-Luc@ama-1 Δlap (*Pb* Δlap). A comparison mutant for *Pb* Δpm4-Δbp2 of a growth-attenuated *P. berghei* lacking leucyl aminopeptidase (LAP) but is not impaired in Hz production (44).

**Figure 1:**
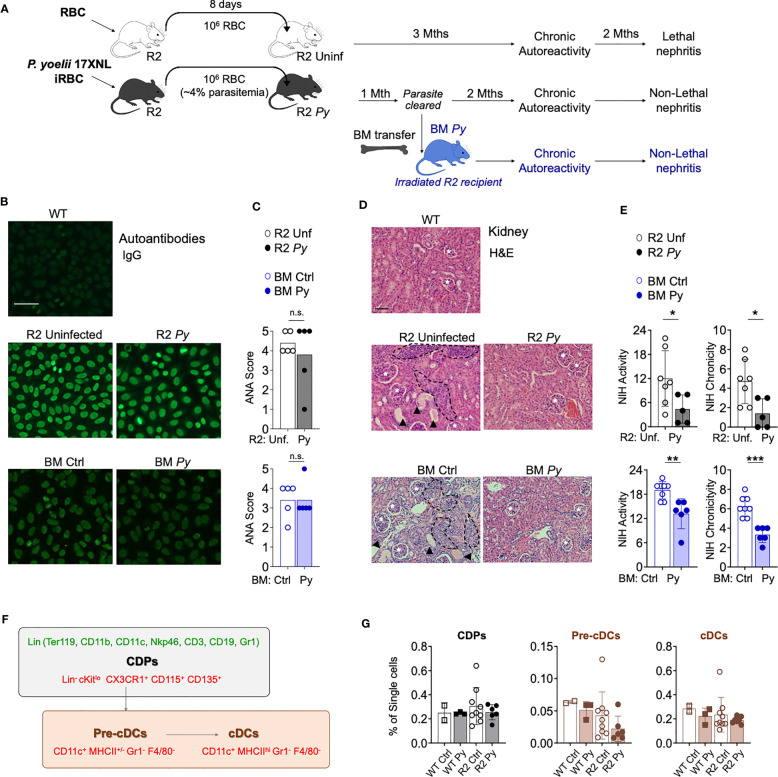
*P. yoelii (Py)* infection and transfer of BM from previously infected mice protects from kidney nephritis without reducing autoreactivity. **(A)** Schematic representation of the experimental injections performed. To ensure equivalent infection rates, seeder R2 mice were injected with frozen aliquots of *Py*-infected or non-infected RBCs. At a point when parasitemia is 4% (usually 8 days after injection), the amount of blood equivalent to one million infected RBCs was injected i.p into experimental mice. Control mice received the same amount of blood from uninfected mice. After the infection is cleared, long-term lupus symptoms were measured (autoantibodies and kidney nephritis). For BM transfer, we extracted BM cells from R2 mice that were infected or uninfected (1-month post-infection, without presence of parasite) and transferred into R2 mice. **(B)** Representative images of IgG serum autoantibodies using the ANA test. WT refers to C57Bl/6J mice. Serum from experimental mice was incubated over Hep-2 cells and then stained with Goat anti-mouse IgG Alexa 488. Scale bar 50 µm. **(C)** Anti-nuclear antibodies (ANA) in serum 4 months after transfer. Scores were defined by the positivity for different serum dilutions: ANA 1 (1:100), ANA 2 (1:300), ANA 3 (1:900), ANA 4 (1:2700), ANA 5 (1:8100). Positive (score 5, R2 mice) and negative (Score 0, WT mice) controls were used to define the scores. Data are represented as mean for 5 mice per group, n.s; non-significant. **(D)** Representative images of kidney sections stained with Hematoxylin and Eosin (H&E) autoantibodies from 3 independent experiments. Glomerulus (white stars), infiltrated areas (dashed black lines) and protein deposits (black arrowheads) are indicated. Scale bar 100 µm. **(E)** NIH activity and chronicity indexes calculated as described in methods section. Detailed scoring shown in **Figure S1**. Data are represented as the mean ± SD from 5 mice per group. Student t-test *p<0.05, **p<0.01, ***p<0.001. **(F)** Scheme showing various populations in the BM DC lineage. Markers used for identification of individual populations by flow cytometry were colored in green (lineage markers) or in red (gating strategy). Common DC precursors (CDP, c-Kit^lo^) were identified by the lack of lineage markers (Ter119, CD11b, CD11c, Nkp46, CD3, CD19 and Gr1) and the expression of CX3CR1, CD115 and CD135. Conventional DC (cDC) stages were distinguished by the high (Pre-cDCs) or low (cDCs) expression of MHC-II among the CD11c^+^Gr1^-^F4/80^-^ population. **(G)** Graphical representation of frequencies BM populations quantified by flow cytometry in WT and R2 mice 4 months after infection. Data are representative of two separate experiments, one of them shown indicated in the graph as the mean ± SD from 2 (WT Ctrl), 3 (WT Py), 9 (R2 Ctrl) and more than 6 (R2 Py) mice per group. Pair comparison analysis denoted no significant differences between infected and uninfected samples in each genotype group.

For all the parasites used, R2 mice were injected intraperitoneally (i.p.) with the amount of blood collected from an infected mouse (3-4% parasitemia) that would contain 10^6^ *Plasmodium*-infected Red Blood Cells (iRBC). For uninfected mice, the amount of blood equivalent to one million infected RBCs was injected. Details of the experimental design are described in **Figure 1A**. Parasitemia was measured by flow cytometry as the percentage of iRBCs (DAPI^+^ Ter119^+^) out of the total RBCs (Ter119^+^) acquired from peripheral blood.

### Bone marrow transfer experiments

BM of R2 infected or non-infected mice were transferred one month after infection into lethally irradiated R2 mice of 2 months of age. Recipient mice were irradiated at 940 rads the day before. BM cells were extracted from the bone by high-speed centrifugation and 20 million cells were re-injected intravenously (i.v.) into recipient mice in 200μl of PBS.

### Hemozoin detection and isolation

Hz crystals were detected by light microscopy from tissue sections or smears as a black colored pigment spots. Also, Hz deposits were detected as black or white dots by differential interference contrast (DIC) and reflection contrast polarized light microscopy (RCM). Semiquantitative analysis of free Hz in the liver or spleen was performed by colorimetric assay using non-infected, *Py*-infected or *Pb*-infected mice. Briefly, organs were dissociated by using gentleMACS™ Octo Dissociator (Miltenyi Biotec) following manufacturer instructions. The dissociated solution was centrifuged 300 g for 5 min and the supernatants were mixed with PBS proportionally to the tissue weight. The obtained solutions were diluted 1/2, 1/4 and 1/8 in PBS-Washing buffer 25% and were read in spectrophotometer at 450 nm and 670 nm.

For Hz isolation, we dissociated spleens from infected animals (1 month after infection) in 3ml of PBS using gentleMACS™ Octo Dissociator (Miltenyi Biotec) following manufacturer instructions. The obtained solution was centrifuged 300 g for 5 minutes and the supernatants were filtered and mixed with 1 ml of autoMACS Pro Washing Solution (Miltenyi Biotec) for 5 min. After adding 2ml of PBS, samples were magnetically processed in the autoMACS® Pro Separator (Miltenyi Biotec) at 4°C using the “PMalaria” program. The obtained Hz-enriched solution was centrifuged at 10,000 rpm and the pellet was resuspended in PBS and stored at -20°C for future injections. All this process was performed in parallel with uninfected controls. Before the injections, Hz suspensions were passed through a syringe and filter with a 70 µm strainer. 2-months old R2 mice were injected i.p. with the equivalent to 1/10 of spleen per mouse. Commercial Hz was obtained from Invivogen (NLRP3 inflammasome inducer, cat# tlrl-hz) and was injected into R2 mice at 0.6 mg/ml in PBS.

### Anti-nuclear autoantibody analysis

The detection and measurement of anti-nuclear autoantibody levels were made using 12-well microscope slides coated with Hep-2 cells (Hep-2 kit, MBL). Serum dilutions were incubated over the Hep-2 cells during 30 min at room temperature (RT). After PBS wash, Alexa Fluor 488 conjugated anti–mouse IgG was added at 0.83 μg/ml in PBS for 30 min at RT. Cells were washed three times with PBS and visualized by fluorescence microscopy. Using a positive (R2 mice) and negative (WT mice) control samples, ANA scores were defined by the fluorescence detection in these serum dilutions: 1 (1:100), 2 (1:300), 3 (1:900), 4 (1:2700), 5 (1:8100).

### Proteinuria

Proteinuria was measured using urinalysis dipsticks (Chemstrip 2GP, Fisher Scientific) and semiquantitatively categorized as 0, <30 mg/dL; 1, 30 mg/dL; 2, 100 mg/dL; 3, 500 mg/d; 4, deceased due to severe proteinuria.

### Tissue preparation and kidney histopathology

Tissues were fixed in formalin and embedded in paraffin for sectioning. Sectioned samples were stained by hematoxylin and eosin (H&E). Kidney H&E sections were analyzed and scored in a blinded way by an expert pathologist (CF.Q) following the National Institutes of Health activity and chronicity indexes revisited by the International Society of Nephrology/Renal Pathology Society classification for lupus nephritis (26). We considered the following characteristics in NIH activity index: endocapillary hypercellularity, neutrophils/karyorrhexis, fibrinoid necrosis, hyaline deposits, cellular/fibrocellular crescents and Interstitial Inflammation. In NIH chronicity index: total glomerulosclerosis score, fibrous crescents, tubular atrophy and interstitial fibrosis were considered.

### *In vitro* analysis of BMDCs

BM cells from WT, infected R2 or uninfected R2 mice were extracted and incubated with GMCSF (25 µg/ml) for 7 days and incubated with spleen-isolated Hz for 5 or 2 more days. Cytokines were measured using BD Cytometric Bead Array (CBA) Mouse Inflammation Kit (cat #552364) following the manufacturer instructions. Data were acquired in LSRII cytometers (BD Biosciences), and the data were analyzed using FCAP software (BD biosciences). Total cell numbers were determined and percentage of live DCs (Live/Dead^-^CD11b^+^CD11c^+^) were measured by flow cytometry at day 12.

### Flow cytometry staining

Single cell suspensions obtained from the BM were incubated 5 min at RT with ACK lysis buffer (Fisher Scientific) to deplete RBCs and filtered with 70 µM strainer. After wash, cells were resuspended in staining solution (PBS containing 2% FBS) and blocked with 0.1 mg/ml of purified rat anti-mouse CD16/CD32 (BD Biosciences) for 15 min at 4°C. Samples were stained with the corresponding conjugated antibodies diluted 1/200 for 30 min at 4°C. If necessary, cells were fixed with PFA 1% in PBS for 30 min, washed and resuspended in PBS. Live and dead cells were discriminated using LIVE/DEAD™ Fixable Yellow Dead Cell Stain Kit (Fisher scientific). Samples were acquired on a CytoFLEX (Beckman Coulter) flow cytometer and then analyzed with FlowJo (TreeStar Technologies) or CytExpert (Beckman Coulter) software.

The anti-mouse antibodies used for flow cytometry were the following: B220-APC-Cy7 (clone RA3-6B2, Biolegend), CD4-PE (clone GK1.5, Biolegend), CD8a-APC (clone 53-6.7, Biolegend), CD11b-FITC NS BV421 (clone M1/70, BD Biosciences), CD11c-PE and PE-Cy7 (clone N418, Biolegend), CD38-APC (clone 90, Biolegend), CD44-PE-Cy7 (clone IM7, Biolegend), CD45.2-APC (clone 104, Biolegend), CD62L-BV421 (clone MEL-14, Biolegend), c-Kit/CD117-BV421 and PerCP-Cy5.5 (clone 2B8, Biolegend), Ly6-C-APC-Cy7 (clone HK1.4, Biolegend), Ly6-G-PE-Cy7 (clone 1A8, BD Biosciences), MHC-II I-A/I-E-Alexa Fluor 488 (clone M5/114.15.2, Biolegend), NK-1.1-Alexa Fluor 700 (clone PK136, BD Biosciences), PDCA-1/BST2/CD317-PE (clone 129C1, Biolegend), TCRb-BV510 and -FITC (clone H57-597, Biolegend and BD Biosciences) and SiglecF-PE (clone 1RNM44N, eBioscience). For lineage exclusion, BV510 conjugated anti-mouse CD3 (clone 17A2), NK-1.1 (clone PK136), B220 (clone RA3-6B2), TER119 (clone TER-119), CD11c (clone N418), CD11b (clone M1/70) and Ly6-G/Ly6-C (Gr-1) (clone RB6-8C5) antibodies were used, all from Biolegend. DC precursors were gated using the following antibodies: CX3CR1-PE (clone SA011F11, Biolegend), SiglecH-BV421 (clone 551, Biolegend), CD115-APC (clone AFS98, Biolegend), CD135-PE (clone A2F10.1, BD Biosciences), Ly6-G/Ly6-C (Gr-1)-APC-Cy7 (clone RB6-8C5, Biolegend), F4/80-APC (clone BM8, Biolegend).

### Quantitative real-time PCR analysis

For gene expression analysis using whole kidney RNA, fresh BM extracts were frozen at -80°C in RNAlater solution (Thermo Fisher Scientific) for future use. Samples were dissociated in RLT buffer with gentleMACS™ Octo Dissociator and the RNA was extracted using RNeasy mini kit (Qiagen). Samples containing 0.5 to 2 µg of RNA were transcribed into cDNA and Genomic DNA was eliminated in pretreatment with RT^2^ First Strand Kit (Qiagen). Quantitative PCR was performed with RT² SYBR Green qPCR Mastermix (Qiagen) and the Cytokines & Chemokines RT^2^ Profiler PCR Array kit (Qiagen) using a CFX Connect Real-Time system and CFX analysis software (Bio-Rad). Data analysis was carried out following the ΔΔCT method using the Qiagen Data Analysis (https://dataanalysis2.qiagen.com/pcr), which calculates fold change/regulation using ΔΔCT method, in which ΔCT is calculated between gene of interest (GOI) and an average of reference genes (HKG), followed by ΔΔCT calculations (Δ CT (Test Group)-Δ CT (Control Group). Fold Change is then calculated using 2^ (-ΔΔCT) formula. Student’s t-test of the replicate 2^(-ΔCT) values for each gene in the control group and treatment groups.

### Statistical analysis

Statistical significance of data was calculated with Prism 7.0 software (GraphPad). For comparisons between two normally distributed groups a two-tailed unpaired t-test was used. Non-parametric data was analyzed using Mann-Whitney U test. For comparison between more than two groups statistical analysis was performed using ANOVA for parametric data and Kruskal-Wallis test for non-parametric data.

## Results

### The protective effect of *P. yoelii* 17XNL (*Py*) on autoimmune nephritis is transferrable by BM cells

We reported before that a self-resolved infection with *Py* early in life protected autoimmune-prone R2 mice from lethal nephritis without diminishing systemic autoimmunity (14). We observed that infection with *Py* reduced infiltration of BM-derived leukocytes into the kidney. The assumption was that one or various BM populations were altered by the parasite in such a way they no longer caused kidney-destroying pathology, and that this phenotype was transferable by reinjecting BM into a new autoimmune-prone recipient mouse. We confirmed the BM transferability with the experiments shown in **Figures 1A-E**. Experimental animals were prepared by injecting *Py*-infected RBCs from seeder mice into recipient R2 mice, while uninfected RBCs were injected in control recipients. Autoimmune symptoms were tracked for up to six months after the injection: circulating IgG anti-nuclear antibodies (ANAs) were measured by Indirect Fluorescent Antibody (IFA) on Hep-2 cells (**Figure 1B**) and kidney disease was detected by histology (**Figure 1D**). Some of the *Py*-infected mice were used as BM donors one month after the injection, when the infection was completely cleared. As shown in **Figures 1B, C**, we observed that systemic autoreactivity progressed in autoimmune-prone mice irrespective of infectious status, since both infected (R2 Py) and uninfected R2 mice were ANA positive. Likewise, autoreactivity was equally present in reconstituted mice receiving BM from *Py*-infected (BM *Py*) or uninfected controls (BM Ctrl) (**Figures 1B, C**). In contrast, the severity of kidney disease (measured by leukocyte infiltration and protein deposits, i.e. NIH activity/chronicity, **Figure S1**) was significantly reduced in infected mice (R2 *Py*) or in those mice receiving infected BM (BM *Py*) compared to the most destructive type of nephritis that was developed in uninfected R2 mice or in those receiving BM from uninfected mice (BM Ctrl) (**Figures 1D, E**, **S1**).

Our previous work pointed to a critical role of DCs in end-stage nephritis developed in R2 mice and prevented by *Py* infection. Thus, we used these new experiments to investigate *Py*-induced alterations of DC precursors that might reduce the output of mature DCs with potential to infiltrate inflamed organs. As shown in the scheme in **Figure 1F**, a common DC precursor (CDP) gives rise to an intermediate cell stage known as the pre-DC, which later develop into mature DCs. We used the flow cytometry strategy shown in **Figure 1F** to determine frequencies of these populations in BM from *Py*-infected mice and compared with samples from uninfected mice (**Figure 1G**). We found no significant changes in these frequencies due to infection, neither in WT nor in lupus-prone R2 mice. We did observe a non-significant tendency of lower numbers of mature DCs in R2 samples compared to non-lupus WT controls, but that seemed independent of infectious status. Overall, this result suggests that the effect of malaria in preventing lupus-associated myeloid cell infiltration in the kidney is not due to a change in the number of precursors of the monocyte-DC lineage in the BM.

### Hz crystals produced during *Py* infection accumulate in spleen, BM and liver but not in kidney

**Figure 2:**
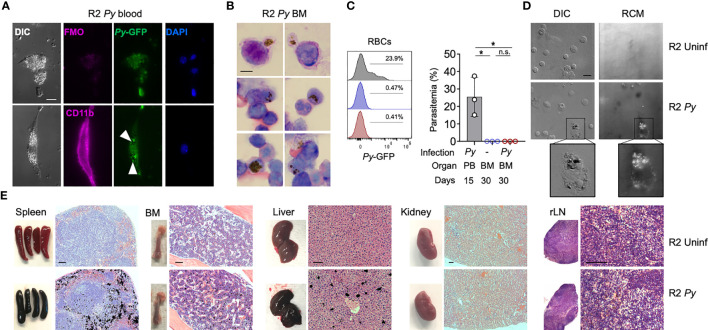
Presence of Hz during and long-term after *Py* infection. **(A)** Fluorescence images showing the presence of malaria parasite (*P. yoelii* 17XNL-GFP, *Py*) and Hz inside peripheral blood myeloid cells during infection (15 days after injection). Myeloid cells (CD11b^+^, pink) containing parasites (GFP^+^, green) and Hz (black spots indicated by white arrows). Nucleus are stained by DAPI. Scale bar 10 µm. **(B)** BM smear stained with Giemsa 1 month after infection. Hz crystals are shown as black spots. Representative images from 3 independent mice. Scale bar 5 µm. **(C)** Analysis of the presence of *Py* parasites in BM at 1 month after infection. Histograms and graph showing the parasitemia levels: percentage of RBC containing parasites (Ter119^+^GFP^+^) out of total RBCs (Ter119^+^). BM analysis 30 days after infection was showing as red and uninfected control as blue. As a positive control peripheral blood from infected mice at day 15 was used (grey). Data are represented as mean ± SD for 3 mice per group. *p,0.05; n.s: non-significant. **(D)** Images showing the presence of pigment inside BM cells 5 months after injection. Hz deposits were detected as white dots by differential interference contrast (DIC) and black dots by reflection contrast polarized light microscopy (RCM). Representative images from 3 independent experiments. Scale bar 10 µm. **(E)** Images of whole organs and organ sections stained with H&E 5 months after infection. Hz deposits can be detected in black in the spleen, BM, liver but not in the kidney and LNs. Representative images from more than 3 independent experiments. Scale bar 100 µm.

*Py* is normally cleared by the host response in about 20 days, but it may leave behind products from its metabolism that can persist in some organs for long periods of time, an effect that has been described in infections with other malaria parasites (28). For example, Hz produced and accumulated with *Py* infection could alter the functionality of erythrophagocytic cells. This alone could explain long-term BM alterations we observe in infected R2 mice. To investigate this possibility, we employed various techniques to detect Hz in tissues at different timepoints after *Py* infection of autoimmune mice. During infection (day 15), fluorescence microscopy showed peripheral blood myeloid cells engulfing live GFP^+^ parasites with Hz accumulation (**Figure 2A**). Early after infection (one month), Giemsa staining of BM smears revealed the presence of Hz inside leukocytes (**Figure 2B**). We confirmed the absence of infection at that stage by flow cytometry (**Figure 2C**). We detected Hz in BM cells 5 months post-infection with the use of reflection contrast polarized light microscopy (RCM) (47) (**Figure 2D**). Finally, we examined several organs by histology searching for Hz crystal deposition months after infection and observed that Hz accumulated in spleen, BM and liver, but not in kidney or renal lymph nodes (**Figures 2E**, **S2**). These results revealed that BM cells had internalized Hz during and after the infection and that Hz accumulates in several organs up to 5 months after infection. Interestingly, kidney was the one organ free of Hz accumulation at any stage, even though it was uniquely altered in the subsequent progression of autoimmune pathology.

### Isolated Hz does not replicate the kidney-protective effect of *Py* infection

**Figure 3:**
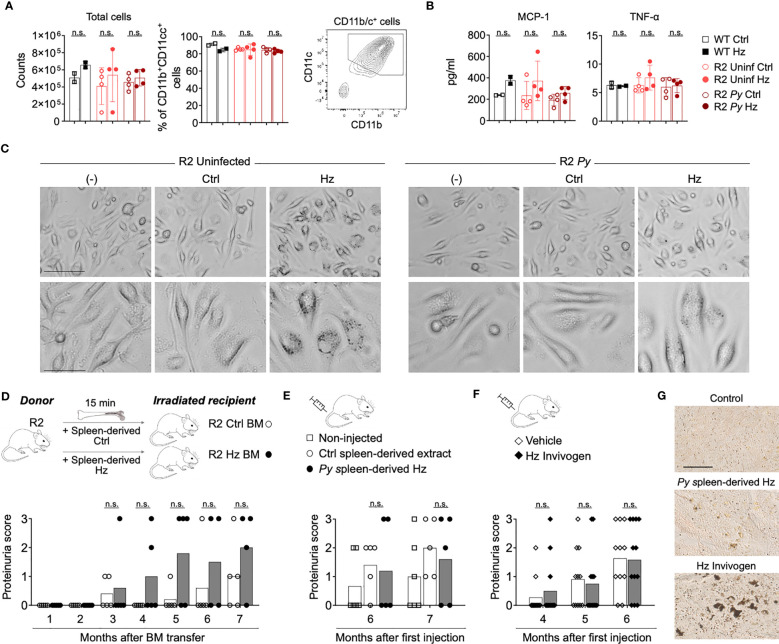
Isolated Hz does not alter BMDCs development and does not protect from lupus nephritis. **(A-C)** BM cells from WT C57Bl/6J mice (black) and infected (dark red) or uninfected (light red) R2 mice (at 1-month post-infection) were extracted and cultured to obtain BMDCs. BM cells were incubated with GMCSF (25 µg/ml) for 7 days and incubated with spleen-isolated Hz for 5 more days. The Hz was isolated from the spleen from uninfected (Ctrl) or infected mice (spleen-derived Hz) by magnetic separation. **(A)** Total cell numbers and percentage of DCs (Live/Dead^-^CD11b^+^CD11c^+^MHC-II^+^) acquired by flow cytometry at day 12. Data are represented as the mean ± SD of 2, 4 and 4 mice per group. n.s; non-significant. **(B)** Levels of inflammatory cytokines MCP-1 and TNF-α in the supernatant at day 12. **(C)** Representative images showing the BM-derived DCs in presence or absence of Hz at day 9 in BMDCs incubated with media only (-) or media with spleen-derived extract form uninfected (Ctrl) or infected mice (Hz). Bar scale corresponds to 100 µm (upper row) and 40 µm (bottom row). **(D)** Transfer of BM cells pre-incubated with Hz. 2 months old R2 recipient mice received BM cells that were pre-incubated with spleen-derived Hz or Ctrl for 15 minutes at 37°C. Graph shows the proteinuria score monthly after transfer. The score was defined as 1, 30mg/dL; 2, 100mg/dL; 3, 500mg/dL of protein in urine. Data are presented as the mean of 5 mice per group. **(E, F)** Hz injections: 2 months old R2 mice were injected with spleen-derived **(E)** or commercial **(F)** Hz at different timepoints, then the proteinuria was analyzed. **(E)** Spleen-derived extract from uninfected or infected R2 mice was injected i.p. once per week (8 times) and at month 4 and 6. The amount injected was equivalent to 1/10 of spleen per mouse and per injection. Proteinuria scores were measured at month 6 and 7 after first injection. Data are presented as the mean of 6, 5 and 5 mice per group from two independent experiments. **(F)** 0.6 mg/ml of commercial Hz (Invivogen, tlrl-hz) or DMSO control (vehicle) was injected 8 times i.p. Proteinuria scores were measured at month 4, 5 and 6 after first injection. Data are represented as the mean of 11 and 12 mice per group from two independent experiments. n.s; non-significant. **(G)** Representative images of unstained spleen sections from mice injected with spleen-derived Hz or commercial Hz, corresponding to **(E, F)** Scale bar 50 µm.

Several reports have suggested that accumulated Hz crystals are immunomodulatory (28–30), a possible rationale for the *Py*-mediated BM alteration in autoimmune-prone mice. To investigate this, we performed *in vitro* and *in vivo* studies with Hz that we purified from the spleen of *Py*-infected R2 mice. Because our previous studies pointed towards DCs as likely targets of the parasite protective effect on autoimmune disease, we incubated BM-derived DCs (BMDCs) in the presence of isolated Hz and analyzed its impact. Incubation with Hz for 5 days did not alter DC numbers or MCP-1/TNF-α production significantly (**Figures 3A, B**). We did not detect significant levels of IL-6, IL-10, IFN-γ and IL-12p70 (data not shown). Similar results were obtained with reduced incubation time (**Figure S3**). Phase contrast microscopy revealed that BMDCs did indeed associate with or internalize the added purified Hz while their morphology remained normal (**Figure 3C**).

We next examined the effect of Hz on the progression of autoimmune disease *in vivo*. BM cells from R2 mice were incubated in the presence or absence of that we had extracted from spleen of infected mice (Spleen-derived Hz), then these cells were adoptively transferred into lethally irradiated R2 mice. The results revealed that the incubation of donor BM with Hz did not change the kidney disease in recipient mice compared with those receiving control BM (**Figure 3D**). In addition, we directly injected our own isolated Spleen-derived Hz or commercially obtained Hz multiple times into autoimmune mice and observed no reduction in proteinuria score, indicating that this type of Hz preparation does not confer protection from end-stage nephritis (**Figures 3E, F**). We verified by histology that there was accumulation of Hz in spleen and BM of mice receiving injections of Hz preparations (**Figures 3G**, **S3C**).

### Infection with a Hz-restricted *P. berghei ANKA* line is self-resolved

**Figure 4:**
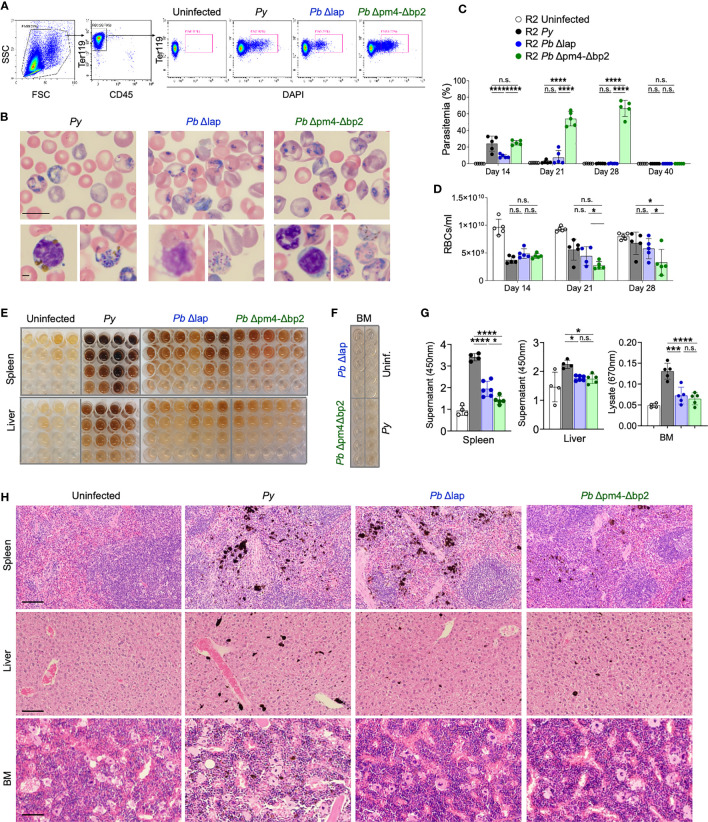
P*. berghei* ANKA mutants produce and accumulate less Hz crystals and achieve similar self-resolved infective process *as P. yoelii*. **(A)** Flow cytometry gating strategy to determine the percentage of iRBCs (Parasitemia) in *P. yoelii* 17XNL *(Py*, black), *P. berghei* ANKA Δlap (*Pb* Δlap, blue) and *P. berghei* ANKA Δpm4-Δbp2 (*Pb* Δpm4-Δbp2, green). **(B)** Representative pictures of blood smears stained with Giemsa 14 days after infection. Scale bar 10 µm (top) and 2 µm (bottom). **(C, D)** Parasitemia percentage **(C)** and RBCs/ml **(D)** in peripheral blood at the indicated days after infection. Data are presented as the mean ± SD 5 mice per group. One-way ANOVA analysis was performed. *p<0.05, ***p<0.001, ****p<0.0001, n.s; non-significant. **(E)** Images of organ supernatants serially diluted in 3:1 PBS:washing buffer: 1/2 (row 2), 1/4 (row 3) and 1/8 (row 4) dilution. Columns represent individual mice. **(F)** Image of undiluted BM extracts from the indicated condition (infected with specific parasite or uninfected). Each well originates from an individual animal. **(G)** Quantification of free pigment from tissue supernatants at 450 nm at 1/2 dilution or undiluted extract at 670nm from BM. Data are presented as the mean ± SD from 4, 4, 6 and 5 mice per group and representative from 2 independent experiments. One-way ANOVA analysis was performed. *p<0.05, n.s; non-significant. **(H)** Tissue sections from spleen, liver and BM stained with H&E. Images were taken with 20x objective (100 µm bar scale). Pigment Hz deposits can be detected by its black color.

Because purified Hz might not behave exactly as Hz produced *in vivo* by the parasite, a better assessment of its role required the use of a parasite line with impaired Hz production. We therefore selected mutant *P. berghei* ANKA (*Pb*) line (*Pb* Δpm4-Δbp2) which lack expression of enzymes plasmepsin 4 (PM4) and berghepain-2 (BP2). Blood stage parasites of this mutant line show a strongly reduced hemoglobin digestion and Hz formation. *Pb* Δpm4-Δbp2 blood stage parasite have a reduced growth and multiplication rate in mice, but infections achieve high levels of parasitemia with time. For control, another *P. berghei* ANKA mutant line (*Pb* Δlap) has been generated that shows a similar slow growth profile of blood stage parasites but produces Hz levels comparable to that of wild type *P. berghei* ANKA parasites (44). Previous studies with these mutant lines did not explore effects beyond 60 days or resolution of the infection in the absence of antimalarial treatments in animals with chronic disease. Thus, we initiated our studies by examining the behavior of *Pb* mutant parasites in long term experiments and then comparing them with our documented *Py* infection in autoimmune mice, with the same approach as described in **Figure 1**. Parasitemia was measured by the percentage of infected RBCs in peripheral blood by flow cytometry (**Figures 4A, C**) or by counting Giemsa-stained blood smears at day 14, 21, 28 and 40 after parasite injection (**Figure 4B**). The total number of RBC/mL was determined in parallel to assess the impact of infection on blood counts (**Figure 4D**). We determined that both *Pb* parasite lines instigated a self-resolved infection in R2 mice that was cleared by day 40. *Pb* Δlap mutant developed a comparable infection pattern as *Py*: both lines reached similar maximum levels of parasitemia (up to 30%) and extent of anemia (RBC/mL) during the same period, and infections with both lines were cleared between 21 and 28 days (**Figures 4C, D**). Infections with *Pb* Δpm4-Δbp2 line displayed delayed clearance with exceptionally high parasitemia levels (up to 60%) (**Figure 4C**). Even with this level of spread, infection with *Pb* Δpm4-Δbp2 was not lethal, perhaps because this parasite line did not trigger anemia to life-threatening levels (**Figure 4D**). In addition, we detected parasites in circulating reticulocytes and RBCs in infections with all three parasite lines (**Figure S4A**). The number of mature reticulocytes and RBCs was reduced in both BM and blood from infected mice, but that effect was temporary and normal counts were found by day 56 or earlier (**Figures S4B, C**). Low RBC count was not correlated with any protection from lupus nephritis, as this is a regular symptom in lupus mouse models. We confirmed this point by testing hemoglobin and observing that uninfected lupus-prone mice had even more extreme reduction of hemoglobin levels in late stage of disease than mice previously infected with Plasmodium (**Figure S4D**).

We then determined the accumulation of Hz long after the infection of autoimmune mice with the *Pb* mutant parasites. We quantified the Hz content in spleen and liver supernatants and in BM extracts at various dilutions by spectrophotometry (**Figures 4E–G**) (48). In general, mice infected with the *Pb* lines accumulated less Hz in spleen, liver and BM compared with mice infected with *Py* parasites. Comparing within the two *Pb* lines, Δlap accumulated marginally higher levels of Hz in spleen than *Pb* Δpm4-Δbp2 (**Figure 4G**). Spleen had the highest levels of accumulation, particularly compared to BM lysates, which by spectrophotometry showed minimal Hz pigment except for the *Py* sample. This was confirmed in tissue sections stained with H&E, revealing a reduction in Hz deposits months after infection with *Pb* Δpm4-Δbp2 compared with the *Py* infection (**Figure 4H**). Overall, these results demonstrate that mice infected with the Hz-restricted parasite line develop a self-resolved infection with similar or higher parasitemia than *Py* but accumulate lower levels of Hz in the long-term.

### Hz-restricted parasite protects from severe autoimmune nephritis without decreasing autoreactivity

**Figure 5:**
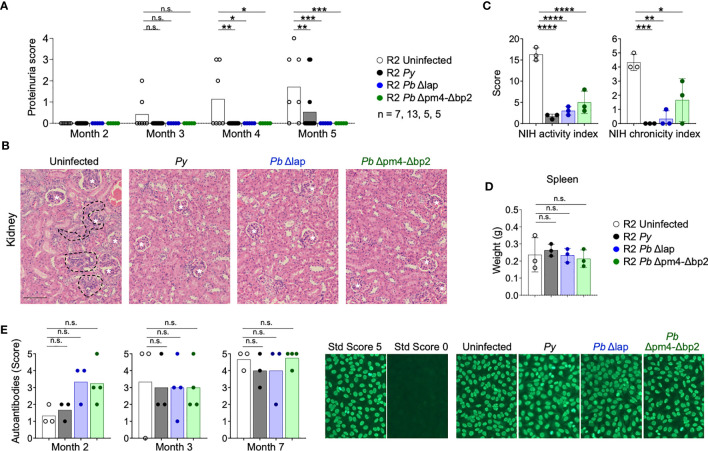
P*. berghei* ANKA mutants protect R2 mice from lethal glomerulonephritis without altering systemic autoimmunity. Study of lupus disease in R2 mice after infection with *P. yoelii* 17XNL *(Py*, black), *P. berghei* ANKA Δlap (*Pb* Δlap, blue) and *P. berghei* ANKA Δpm4-Δbp2 (*Pb* Δpm4-Δbp2, green). **(A)** Proteinuria score monthly after transfer. The score was defined as 1, 30mg/dL; 2, 100 mg/dL; 3, 500 mg/dL of protein in urine. Data are represented as the mean of proteinuria score from 7, 13, 5 and 5 mice per group. One-way ANOVA analysis was performed. *p<0.05, **p<0.01, ***p<0.001, n.s; non-significant. **(B)** Representative kidney tissue sections stained with H&E 5 months after infection. Glomerulus (white stars) and infiltrated areas (dashed lines) are indicated. Scale bar 100 µm. **(C)** NIH activity and chronicity indexes calculated as described in methods section and **supplementary material**. Data are represented as the mean ± SD from 3 mice per group. One-way ANOVA analysis was performed. *p<0.05, **p<0.01, ***p<0.001,****p<0.0001. **(D)** Spleen weight 6 months after infection. Data are represented as mean ± SD from 3 mice per group. One-way ANOVA analysis was performed. n.s; non-significant. **(E)** Autoantibody detection in serum at indicated times. The autoantibody scores were defined by the positive IgG staining in different serum dilutions: 1 (1:100), 2 (1:300), 3 (1:900), 4 (1:2700), 5 (1:8100). Representative images of the immunofluorescence assay (right). Positive (score 5, R2 mice) and negative (Score 0, WT mice) controls were used to define the scores. One-way ANOVA analysis was performed. n.s; non-significant.

We infected autoimmune R2 mice with the Hz-restricted *Pb* mutant lines (*Pb* Δpm4-Δbp2 and *Pb* Δlap) to determine its influence on long-term autoimmune progression. Another experimental set was infected with *Py* parasites so we could establish a proper comparison with our previous data. Infection with all three *Plasmodium* lines, independent of the level of Hz accumulation, had a protective effect on kidney disease measured by proteinuria, nephritis activity or chronicity index upon histological analysis (**Figures 5A-C**, **S5**). Images revealed a significant reduction of nephritic damage in infected mice compared to control. The features of severe pathology that had differential presentation included: glomerular sclerosis, tubular damage and interstitial cell infiltration (**Figure S5**).

As was the case in *Py* infection, the Hz-restricted *Pb* parasite lines Δpm4-Δbp2 and Δlap induced a kidney protective effect on autoimmune mice that did not reduce overall autoreactivity and systemic inflammation. Spleen size (**Figure 5D**) and serum ANAs (**Figure 5E**) were not significantly reduced after infection with *Py* and *Pb* mutants. Thus, *Plasmodium* infection protects from autoimmune nephritis by a process not correlated with the level of accumulation of Hz.

### Infection with *Plasmodium* alters the BM cytokine profile long after infection

**Figure 6:**
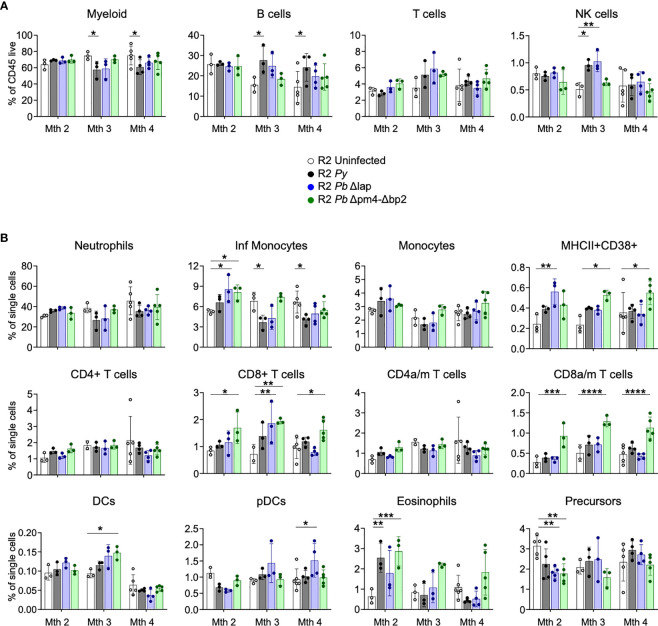
BM leukocyte populations long-term after *Plasmodium* infection. Study of BM leukocytes at 2-, 3- and 4-months post-infection with *P. yoelii* 17XNL *(Py*, black), *P. berghei* ANKA Δlap (*Pb* Δlap, blue) and *P. berghei* ANKA Δpm4-Δbp2 (*Pb* Δpm4-Δbp2, green). **(A)** Percentage of general leukocyte populations out of CD45+ cells: myeloid cells (CD11b^+^), B cells (B220^+^), T cells (TCRβ^+^NK1.1^-^) and NK cells (TCRβ-NK1.1^+^). **(B)** Frequency out of single cells acquired by flow cytometry: neutrophils (CD11b^+^Ly6C^int^Ly6G^+^), inflammatory monocytes (CD11b^+^Ly6C^hi^Ly6G^-^), monocytes (CD11b^+^Ly6C^+^Ly6G^-^), MHC-II^+^CD38^+^ myeloid population, CD4/CD8 activated-memory (a/m) T cells (CD44^+^CD62L^-^), DCs (CD11c^hi^MHC-II^+^), plasmacytoid DCs (pDCs, PDCA-1^+^B220^+^), eosinophils (CD11b^+^SiglecF^+^SSC^hi^) and hematopoietic precursors (Lin^-^c-Kit^+^). Results show representative data (3 and 5 mice per group) of two independent experiments. Data are represented as mean ± SD. One-way ANOVA. *p<0.05, **p<0.01, ***p<0.001, ****p<0.0001. n.s; non-significant results are not indicated.

Since we associated the protective effect of the parasite on autoimmune pathology with its effect on BM cells (**Figure 1**) (14), we examined numbers and activation status of BM cell populations at various timepoints after infection with both *Py* and *Pb* lines. Our results revealed the absence of any persistent changes in the frequencies of leukocyte populations that was common to all three parasite lines (**Figure 6**). We found a slight reduction of myeloid cells mostly due to a reduction of inflammatory monocytes compared with uninfected mice at month 3 and 4 post-infection. CD8 T cells, especially when activated, were significantly increased in infections with the Hz-restricted mutant but unchanged in infection with the other lines, suggesting that this increase is not essential to the kidney protective effect that occurs in infections with all three lines. In addition, there was a transitional reduction of leukocyte precursors and increase of eosinophils at 2 months post-infection (**Figure 6B**). Finally, we found an uneven increase of a CD38^+^ myeloid cell (CD11b^+^, CD11c^lo^) population and of various types of DCs post-infection, suggesting that although frequencies of myeloid cells are similar in every case, their activation status and functionality changed over time (**Figures 6A, B**).

**Figure 7:**
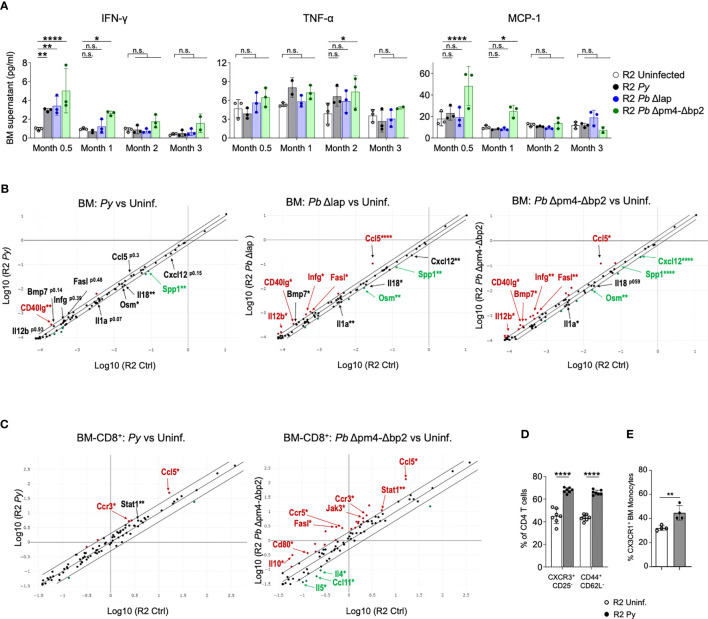
Long term alteration of cytokine/chemokine expression in BM from mice infected with *Plasmodium*. **(A)** Levels of IFN-γ, TNF-α and MCP-1 in BM supernatant of R2 mice at the indicated times post-infection with *P. yoelii* 17XNL *(Py*, black), *P. berghei* ANKA Δlap (*Pb* Δlap, blue) and *P. berghei* ANKA Δpm4-Δbp2 (*Pb* Δpm4-Δbp2, green). BM was incubated in 100µl of PBS for 20 minutes at 4°C, and the supernatant cytokines were measured using BD™ Cytometric Bead Array (CBA, BD biosciences). Data are represented as the mean ± SD from 3 mice per group. Two-way ANOVA. *p<0.05, **p<0.01, ****p<0.0001, n.s; non-significant. **(B)** RNA expression of cytokines and chemokines in BM cells at 2 months after infection with the parasite indicated above the graph using RT² Profiler™ PCR Array Mouse Cytokines & Chemokines (Qiagen). Each graph compares samples from infected mice relative to uninfected controls. Differentially expressed genes found in all three comparisons are indicated with arrows. Red color indicates that genes are increased in infected mice compared to uninfected with more than 1.5-fold change, green color represents the genes reduced more than 1.5-fold and the black genes with asterisk are those with significant differences but in the lower range between 1.25 to 1.5-fold compared to uninfected mice. Cutoff 35 cycles. 4 mice were analyzed per group. p-values are calculated based on a Student’s t-test of the replicate 2^(- Delta CT) values for each gene in the control group and treatment groups. Asterisks represents p values as detailed in **(A)**. Full data is shown in Table format in **Figure S7A**. **(C)** RNA expression of cytokines and chemokines in CD8^+^ cells purified from BM cells at 6 weeks after infection with the parasite indicated above the graph using RT² Profiler™ PCR Array Mouse Th1/Th2 Cytokines & Chemokines (Qiagen). Red/green color indicates that genes are increased/decreased in infected mice compared to uninfected with more than 2-fold change Full data is shown in Table format in **Figure S8**. **(D)** Graphs showing the percentage of CXCR3+CD25- and CD44+CD62L- of the BM CD4+ T cells (CD3^+^) measured by flow cytometry on BM cells of the indicated genotype and condition. Data are represented as the mean ± SD from 7 mice per group. Two-way ANOVA. ****p<0.0001. **(E)** Graph showing the percentage of CX3CR1^+^ cells from the BM Monocytes (CD115^+^CD11b^+^ CD19^-^ CD3^-^ Ly6G^-^ Ter119^-^) by flow cytometry. Data are represented as the mean ± SD from 3 mice per group. T-Test **p<0.01.

As a measure of activation status of BM cells, we tested their cytokine and chemokine expression at various points after the infection. We measured 6 common inflammatory cytokines (IFN-γ, IL-6, IL-10, IL-12p70, TNF-α and MCP-1) in supernatants from BM cell suspensions, which showed an initial increase of IFN-γ and MCP-1 levels during and shortly after infection, particularly evident in R2 mice infected with Hz-restricted parasite (*Pb* Δpm4-Δbp2) (**Figure 7A**). TNF-α was equally expressed at 2- and 3- months post-infection with all three parasite lines (**Figure 7A**). The levels of IL-6, IL-10 and IL-12p70 were low and not significantly different in BM supernatants during and after the infection (**Figure S6**).

For a detailed view of the cytokine profile after infection, we employed a Cytokine & Chemokine RT-PCR Array (Qiagen) on BM RNA from mice infected with the three parasite lines two months after infection and from uninfected control mice (**Figures 7B**, **S7**). Looking for commonalities among all three infections, *Cd40lg* was the only gene significantly upregulated in all three compared to uninfected samples, while there were two genes significantly downregulated in all infected samples: *Spp1* and *Osm1*. A few genes were significantly different in *Pb* Δpm4-Δ*bp2 and Pb* Δlap, while also close to significant (p<0.15) in *Py* samples: *Bmp7* (upregulated), *Il1a* and *Cxcl12* (downregulated). Signs of a robust Th1 immune activation were evident in the two *Pb* samples, with significant upregulation of *Ifng, Il12b, Ccl5 and Fasl*. Bias towards a Th1 response is consistent with the observed high levels of *Cd40lg*, which is strongly upregulated in activated CD8 cells (49). On the other hand, downregulated genes *Il18* and *Il1a* are both expressed downstream of inflammasome activation in macrophages and monocytes (50). All downregulated genes (*Ssp1, Osm, Cxcl12, Il18, Il1a*) have been associated with fibrosis and kidney damage (51–54). *Bmp7*, found upregulated in infected samples, has been described as a stromal cell-derived cytokine that antagonizes fibrosis and can reverse chronic kidney disease (55).

In our previous studies with this mouse model, we found that CCL17, a chemokine expressed by DCs, was integral in the mechanism of the kidney pathology (14). When analyzing chemokines expressed in BM, we detected very low levels of Ccl17 in BM samples, consistent with the low frequency of mature DCs in BM. Only the sample from mice infected with mutant *Pb* Δlap showed significant reduction of *Ccl17* in BM compared to uninfected samples (**Figure S7A**). Further analysis failed to detect Ccl17 transcripts in monocyte fractions from BM of either *Py*-infected or uninfected mice (**Figure S7B**).

We then tested the possibility of a persistent Th1 bias in BM of infected mice by analyzing cytokine/chemokine expression data using CD8 cells positively selected from bone marrow six weeks after infection, when we know the parasite is already cleared. We selected CD8 cells for this analysis because they are producers of type 1 cytokines, specially IFN-γ, and because flow cytometry data from **Figure 6** was suggestive of an increased frequency of CD8 cells 3 months after infection, although these changes were not significant across all simples and all timepoints. Using an RNA-based array to detect Th1/Th2 genes and to find commonality with two different parasites, we show that the highly elevated levels of *Ccl5* that were previously observed in whole BM infected samples (**Figure 7B**), is also a hallmark of CD8^+^ cells present in bone marrow from infected mice (**Figure 7C**). CCL5, also known as RANTES, is a chemokine associated with Type I responses and its transcript accumulates in activated/memory CD8 cells (56). In addition, we found upregulation of the CCL5-binding receptor *Ccr3* in CD8 samples purified from bone marrow of infected mice compared to uninfected controls. Moreover, we observed significant upregulation in both infected samples of *Stat1*, a transcription factor induced by IFN-γ (**Figures 7C**, **S8**). Out of the two parasites, *P.bergei* infection induces the stronger immune response, and in this case we also detect significant reduced expression of the Th2-associated factors *Il4*, *Il5* and *Ccl11*/Eotaxin (**Figure 7C**).

Complementing the gene expression data on CD8 cells, we used flow cytometry to detect signs of Th1 bias in bone marrow CD4 cells and myeloid cells. Th1 skewing of CD4 cells is strongly associated with the expression of the chemokine receptor CXCR3 (57, 58), which we found elevated in *Py*-infected samples compared to uninfected controls (**Figure 7D**). In the case of myeloid cells, they preserve high CX3CR1 expression in environments with elevated IFN-γ, and that results in their retention in bone marrow thus preventing their exit towards inflamed sites (59). We found increased expression of CX3CR1 in myeloid cells from *Py-*infected bone marrow compared to uninfected controls (**Figure 7E**). Together, these results point to a lasting activation profile with Th1 bias in multiple populations of the bone marrow after infection with *Plasmodium*.

## Discussion

The immune response against *Plasmodium* is complex, as the parasite incites a strong immediate response but often this confers imperfect protection against repeated infections (60). It has been proposed that the lack of long-term immunity against the parasite is due to an enduring immunomodulatory effect of the infection (8–10). The protective effect of Plasmodium on lupus nephritis in mouse models was first presented decades ago (14) but very few mechanistic studies have focused on this effect in the years since, and even fewer made use of self-resolving infections in the absence of anti-malarial agents. In one case, it was hypothesized that malaria induced polyclonal antibodies in BALB/c mice that could transfer protection into NZB/W lupus prone mice (25). In contrast, our studies with the lupus-prone FcγR2B[KO] strain eliminated this hypothesis as antibody transfer from infected mice could not protect recipient mice (14).

In our model, we find that the impact of *Plasmodium* on the progression of chronic autoimmune disease is specific to the transition to end-stage kidney disease and transferable by bone marrow cells ( (14) and this manuscript). Bone marrow is recognized as a major parasite reservoir in human infection with *Plasmodium vivax*, a parasite with reticulocyte preference (61). In our studies, all three murine parasite lines infect reticulocytes and thus invade the bone marrow. However, we found no evidence of live parasite persisting past the initial weeks after infection. Most notably, bone marrow adoptive transfer experiments showed no expansion of parasite in new hosts when the donor sample was collected at a time point beyond six weeks post infection. An intriguing possibility is that parasite infection via reticulocyte and BM invasion might be a requisite for the type of BM alterations connected to protection from end-stage kidney disease.

Because the protective effect on autoimmune nephritis occurred long after the infection when the parasites are absent, we could explore underlying mechanisms of protection that may depend on ‘infection after-effects’. These included: persistence of Hz in phagocytic cells and organs, or compensatory alterations of immune cell development and maturation.

It has been shown that Hz accumulates in organs for many months after infection (28). When using the non-lethal *Py* strain, we detected accumulation of Hz most prominently in tissues with high presence of erythrophagocytic cells: spleen, BM and liver. Kidney does not accumulate hemozoin possible because phagocytic cells found in kidney tissue are resident macrophages that do not interact with erythrocytes in circulation (62). In our hands, purified Hz preparations and injections are imperfect experimental designs because it is difficult to replicate the crystal structure and location of the physiologically produced pigment. Thus, we relied on *P. berghei* ANKA parasites with loss of function mutations in enzymes implicated in hemoglobin digestion resulting in highly reduced Hz production and accumulation (29, 44, 45). As there is limited knowledge on the long-term infectivity and biology of these parasite mutant lines, we first analyzed infection-characteristics in our model of autoimmune disease. Compared to parasites of the wild type *P. berghei* ANKA strain, which causes lethality when infecting B6 mice, parasites of the mutant lines (*Pb* Δpm4-Δbp2 and *Pb* Δlap) exhibited slower growth and all infections were self-resolving in the absence of antimalarial treatment. *Pb* Δlap was shown to accumulate reduced levels of Hz and the clearance rate was comparable to the non-lethal *Py* strain. In contrast, infection with *Pb* Δpm4-Δbp2 showed a strongly reduced accumulation of Hz even though parasitemia levels reached up to 60% and the clearance rate was much slower than *Pb* Δlap or *Py*. Interestingly, this high parasitemia did not cause lethality, perhaps because infections did not cause extreme anemia. This might be an indication that Hz production alters RBCs in a way that it accelerates erythrophagocytosis.

When the various Hz-deficient and proficient parasites were assessed for their effect on chronic autoimmune disease, we noted that the kidney-protection was neither parasite-strain specific nor correlated with the production of Hz. We took advantage of the three different non-lethal parasite lines to find commonality that could shed light on the mechanism of protection from severe autoimmune nephritis. We focused on compensatory immune effects, particularly in BM, because our previous study pointed towards alterations of BM cells that would prevent further infiltration of pathological cells into the kidney (14). We confirmed this view in our current study because while we did not see sequalae of the infection (such as the presence of the parasite and Hz accumulation) in kidney, it was noticeable in BM. Also consistent with this view was the fact that BM cells obtained from *Plasmodium*-infected mice donors were able to transfer the kidney-protective effect. Experiments that evaluated changes within the DC lineage in BM long after infection revealed that the frequencies of precursor, immature and mature DCs were not affected by *Plasmodium.* In addition, early after infection we observed a reduction of myeloid cells in BM, but this was a temporary effect and variable among different parasite lines.

While immune cell frequencies in BM were not consistently altered, we found that their cytokine/chemokine expression was modified months after the infection and long after clearance of parasite. Commonalities among infection with the various parasite lines suggested a strong Th1 response consistent with previous reports in the literature (63, 64). A Th1 response is also consistent with increased frequency of activated monocytes expressing CD38 and activated/memory CD8 cells in BM, which we find particularly evident after infection with Hz-restricted parasites (**Figure 6**). We have further confirmed the Th1-skewed environment with the detection of Ccl5-producing CD8 cells and Th1-associated markers in both CD4^+^ and CD11^+^ cells in bone marrow of *Plasmodium* infected mice. On the other hand, two of the cytokines we detected at reduced levels in BM from infected samples, IL1a and IL18, are commonly associated with Th1 responses and downstream of inflammasome activation, a process also reported associated with Hz accumulation in spleen (45). The lower expression detected in the cytokine array could reflect a reduction of precursor transcripts that are cleaved during inflammasome activation (49). Interestingly, IL18 and IL1 can be produced both by immune cells and stromal cells, so that their expression pattern could be complex in the BM environment. Three differentially expressed genes: *Osm* (Oncostatin M), *Ssp1* and *Cxcl12* originate from a variety of cells of the BM, such as stromal cells and osteoblasts. Thus, the overall effect of the infection in BM is not only long-lasting but functionally affecting multiple cell populations. It is likely that these alterations not only affect the progression of autoimmune nephritis as we have seen in this report, but also other long-term conditions that require specific maturation states of immune cells to promote or prevent pathologies.

## Data availability statement

The original contributions presented in the study are included in the article/**Supplementary Material**. Further inquiries can be directed to the corresponding author.

## Ethics statement

The animal study was reviewed and approved by NIAID ACUC.

## Author contributions

LA: Conceptualization, Investigation, and Writing-original draft. BS: Mouse technical support. HK, LK, and HW: Experimental support. C-FQ: Pathological review. LM: Conceptualization. CJ: Resources and manuscript editing. SB: Supervision and Writing-review and editing.
