# Supplementary material for: Plasmodium curtails autoimmune nephritis via lasting bone marrow alterations, independent of hemozoin accumulation
Source: Front Immunol. 2023 Jul 19;14:1192819. doi: 10.3389/fimmu.2023.1192819 (PMC10394379; doi:10.3389/fimmu.2023.1192819)
Supplement: Supplementary file 1 [file DataSheet_1.pdf]

	R2 Ctrl							**R2 Py**					BM Ctrl								**BM Py**					
	983	984	989	990	2803	2804	2806	976	977	978	2816	2819	1403	1404	1405	1406	1407	7487	7488	7498	1408	1409	1410	1411	1412	7494
Endocapillary hypercellularity	1	2	3	3	2	2	2	1	2	1	1	1	2	3	2	3	3	3	3	3	2	2	2	2	2	2
karyorrhexis	1	1	2	2	0	0	0	0	1	1	0	0	2	2	2	2	2	2	2	2	2	2	2	2	2	0
Fibrinoid necrosis (x2)	0	1	3	3	1	1	2	0	1	1	0	0	2	2	2	3	2	3	3	3	2	1	1	1	2	0
Hyaline deposits	0	0	2	2	0	0	0	0	0	1	0	0	2	2	2	2	2	2	2	2	2	2	2	2	2	0
Cellular/fibrocellular crescents (x2)	0	0	2	3	2	2	2	1	1	1	0	0	2	2	2	2	3	2	3	2	2	2	2	2	2	1
Interstitial Inflammation	1	1	3	3	3	2	2	1	1	1	0	0	2	3	2	3	3	3	3	3	2	2	1	2	2	2
NIH activity index	***3***	***6***	***20***	***22***	***11***	***10***	***12***	***4***	***8***	***8***	***1***	***1***	***16***	***18***	***16***	***20***	***20***	***20***	***22***	***20***	***16***	***14***	***13***	***14***	***16***	***6***

Total glomerulosclerosis score	1	1	3	3	2	2	2	0	1	1	0	0	2	2	2	3	3	3	3	2	1	2	1	2	2	1
Fibrous crescents	0	0	1	2	1	2	2	0	1	1	0	0	1	2	1	2	2	2	2	2	1	1	1	1	1	0
Tubular atrophy	0	0	2	2	0	0	0	0	0	0	0	0	0	0	0	0	0	1	1	0	0	0	0	0	0	0
Interstitial fibrosis	1	1	1	1	1	1	1	1	1	1	0	0	2	2	2	1	2	1	2	2	1	1	1	1	1	1
NIH chronicity index	***2***	***2***	***7***	***8***	***4***	***5***	***5***	***1***	***3***	***3***	***0***	***0***	***5***	***6***	***5***	***6***	***7***	***7***	***8***	***6***	***3***	***4***	***3***	***4***	***4***	***2***

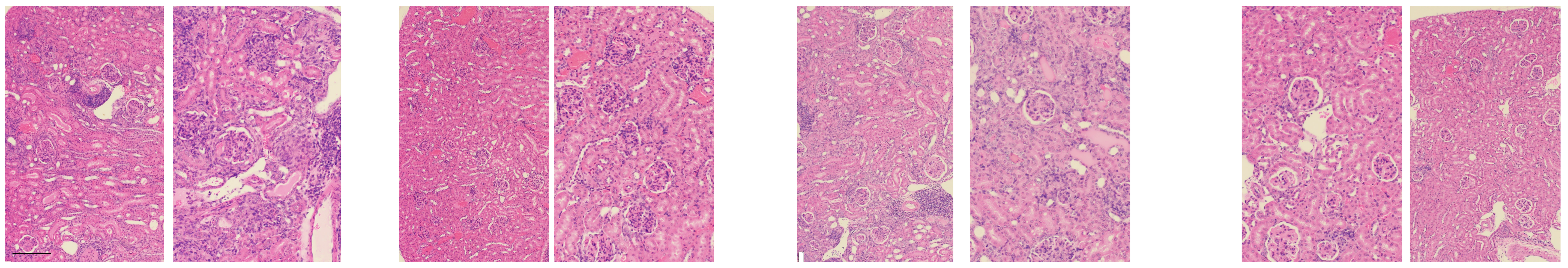

Figure S1. Kidney NIH indexing in R2 mice after infection and after BM transfer.
NIH Activity and Chronicity Index values for every kidney sample studied, with H&E images from each group analyzed. Scale bar 200 µm**.**

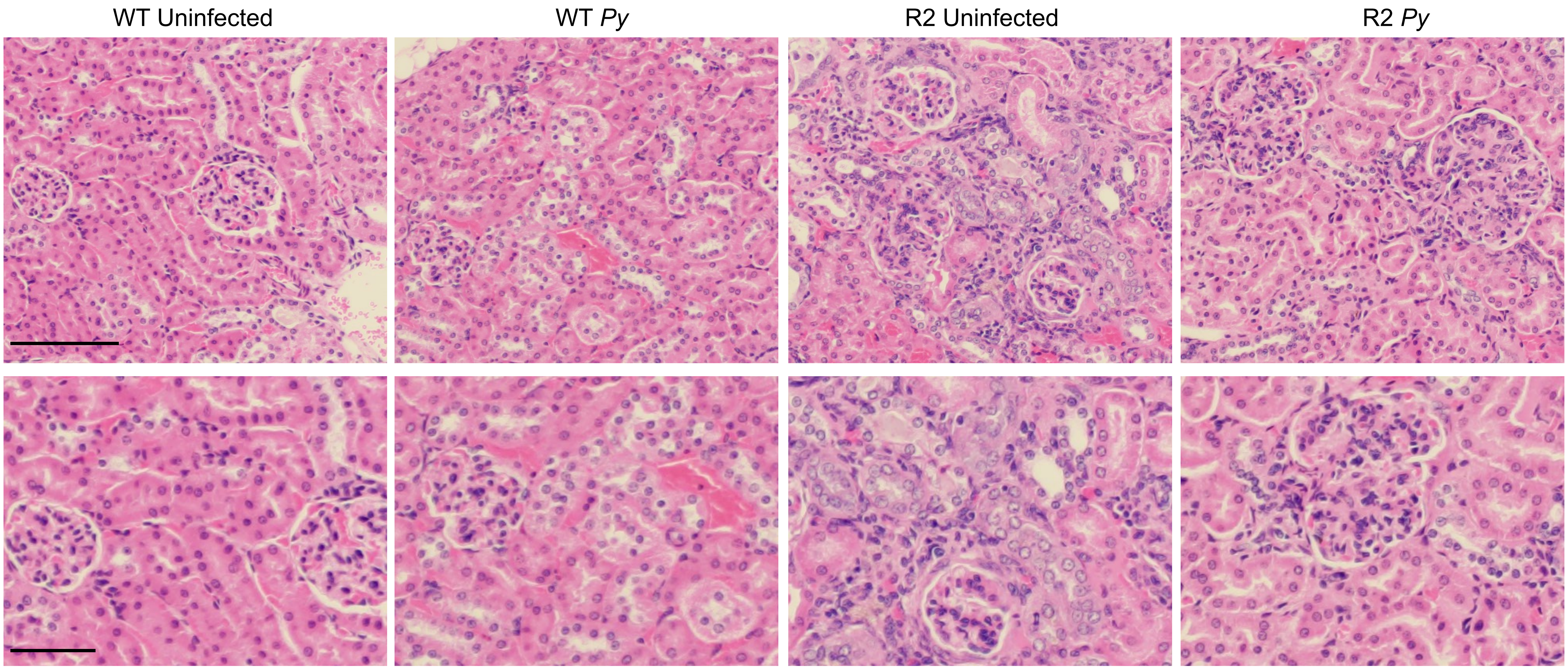

Figure S2. Hz is undetected in kidney tissue long-term after *Py* infection.
Images showing the absence of Hz deposits in kidney sections stained with H&E 5 months after infection. Bar scale corresponds to 100 µm (upper row) and 50 µm (bottom row).

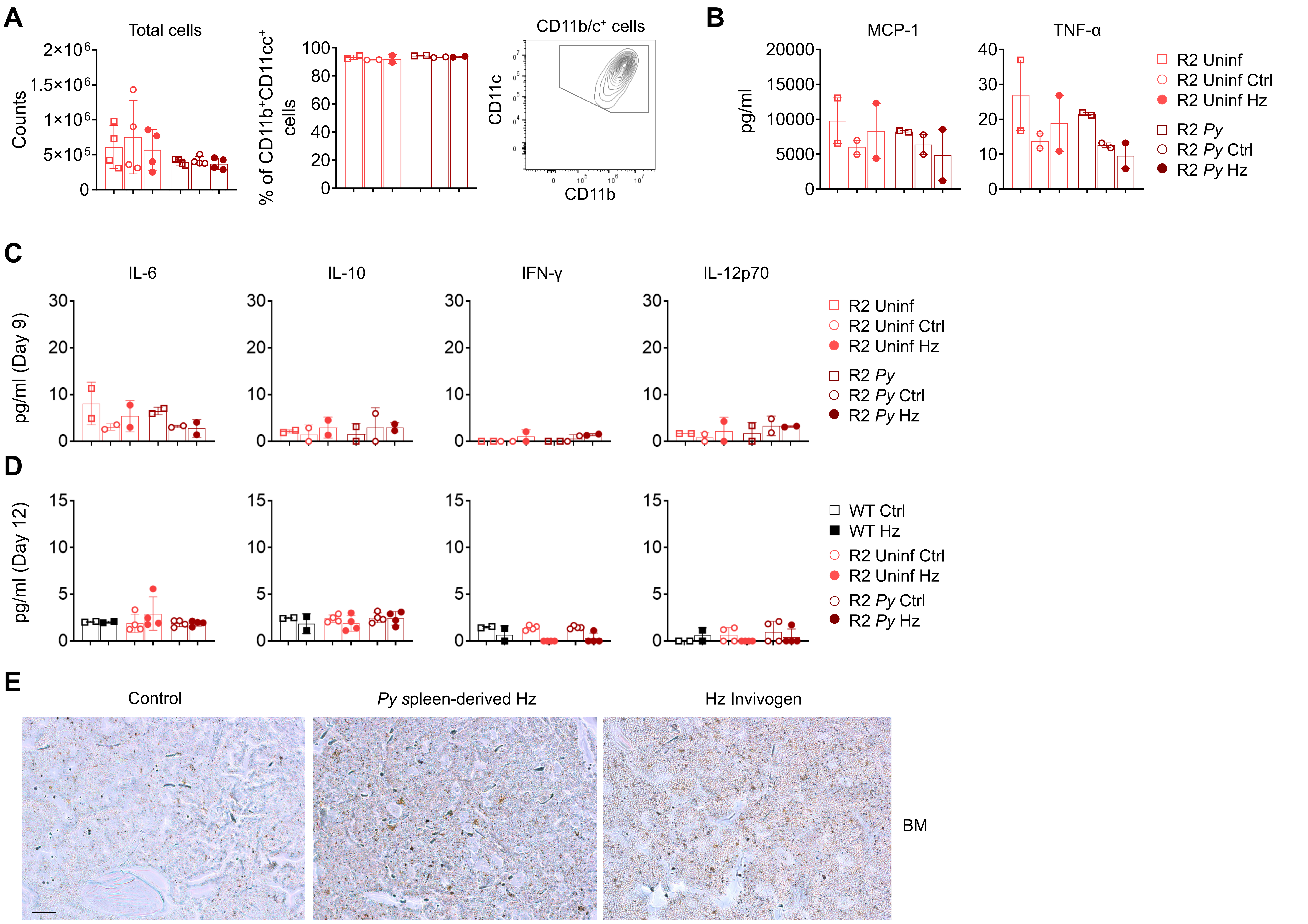

Figure S3. BMDCs develop normally in the presence of Hz.

BM cells from WT and infected or uninfected R2 mice were extracted and cultured at 1 month after infection. BM cells were incubated with GMCSF (25 µg/ml) for 7 days and incubated with spleen-isolated Hz for 2 more days. **A**) Total cell numbers and percentage of DCs (Live/Dead$^-$CD11b$^+$CD11c$^+$MHC-II$^+$) acquired by flow cytometry at day 9. **B**) Levels of inflammatory cytokines MCP-1 and TNF-α in the supernatant at day 9. **C**) Levels of inflammatory cytokines Il-6, Il-10, IFN-γ and IL-12p70 and TNF-α in the supernatant at day 9. **D**) Levels of inflammatory cytokines Il-6, Il-10, IFN-γ and IL-12p70 and TNF-α in the supernatant at day 12. **E**) Representative images of BM sections from mice injected with spleen-derived Hz or commercial Hz, corresponding to Fig 3E and 3F. Scale bar 100 µm.

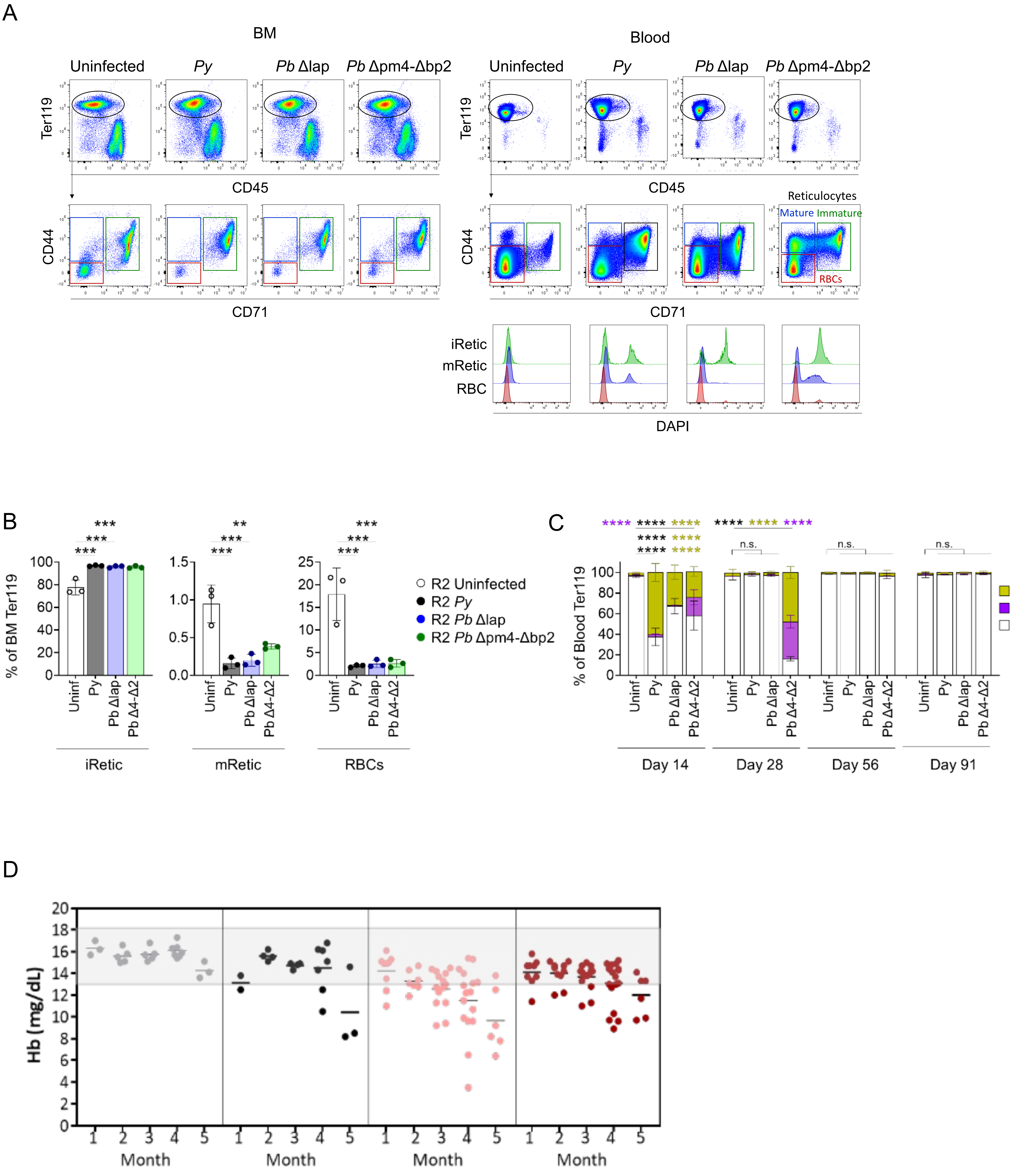

Figure S4. *P. berghei* ANKA infection affects reticulocytes and RBC as in *P. yoelii* XNL infection.

Study of erythroid lineage populations in R2 mice during infection (14 days) with *P. yoelii 17*XNL *(Py)*, *P. berghei* ANKA Δlap (*Pb* Δlap) and *P. berghei* ANKA Δpm4-Δbp2 (*Pb* Δpm4-Δbp2). A) Flow cytometry gating strategy in cells from BM and Blood. Red blood cells (RBCs), immature (iRetic) and mature (mRetic) reticulocytes. Graphs show frequencies of indicated populations in BM (B) and Blood (C) at the indicated times. . Data are represented as the mean ±SD from 3 mice per group. One-way ANOVA. **p<0.01, ***p<0.001, ****p>0.0001. D) Hemoglobin levels in WT (light grey), *Py*-infected WT (black), R2 (pink) and *Py*-infected R2 mice (red).

	Uninfected			*Py*			*Pb* Δlap			*Pb* Δpm4-Δbp2		
	#1601	#1604	#1605	#1614	#1615	#1616	#1619	#1621	#1623	#1626	#1627	#1628
Endocapillary hypercellularity	3	2	3	1	1	1	1	1	1	1	1	2
karyorrhexis	2	2	2	0	0	0	0	0	1	1	0	1
Fibrinoid necrosis (x2)	2	1	1	0	0	0	0	0	0	0	0	0
Hyaline deposits	2	2	2	0	0	0	0	0	0	0	0	1
Cellular/fibrocellular crescents (x2)	2	2	2	0	0	0	0	0	0	0	0	1
Interstitial Inflammation	3	2	3	1	0	1	2	1	2	2	2	2
NIH activity index	***18***	***14***	***16***	***2***	***1***	***2***	***3***	***2***	***4***	***4***	***3***	***8***

Total glomerulosclerosis score	3	2	2	0	0	0	0	0	1	2	0	2
Fibrous crescents	1	1	1	0	0	0	0	0	0	0	0	1
Tubular atrophy	0	0	0	0	0	0	0	0	0	0	0	0
Interstitial fibrosis	1	1	1	0	0	0	0	0	0	0	0	0
NIH chronicity index	***5***	***4***	***4***	***0***	***0***	***0***	***0***	***0***	***1***	***2***	***0***	***3***

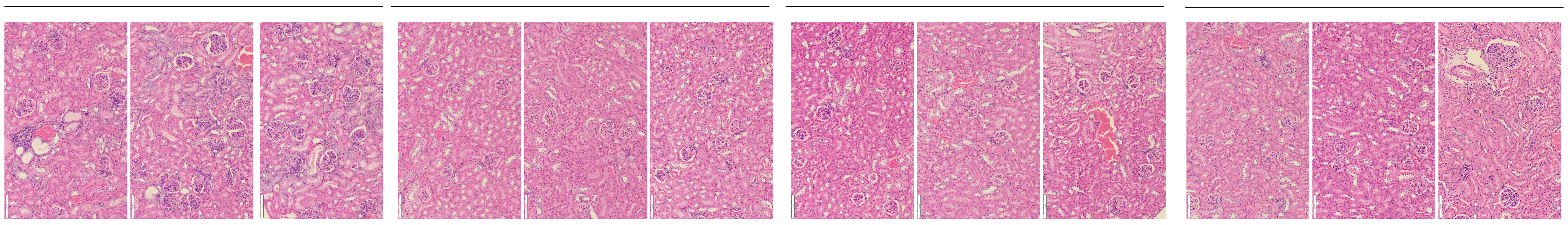

Figure S5. Kidney NIH indexing in R2 mice after infection with *Plasmodium*.
NIH Activity and Chronicity Index values for every kidney sample studied, with H&E images from each group analyzed. Scale bar 100 µm.

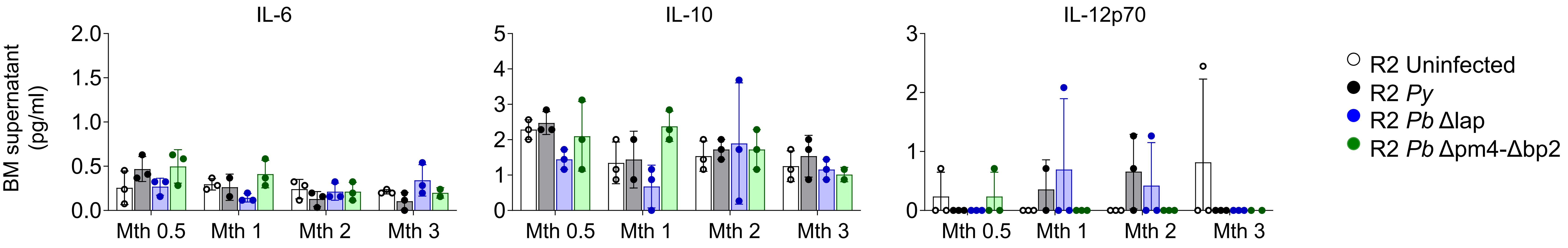

Figure S6. Low levels of IL-6, IL-10 or IL-12p70 are detected in supernatants from BM of infected and uninfected R2 mice.
Levels of IL-6, IL-10 or IL-12p70 in BM supernatant of R2 mice at the indicated times post-infection with *P. yoelii* 17XNL *(Py,* black), *P. berghei* ANKA Δlap (*Pb* Δlap, blue) and *P. berghei* ANKA Δpm4-Δbp2 (*Pb* Δpm4-Δbp2, green).

A

	R2 Py		R2 Pb Δlap		R2 Pb Δpm4-Δbp2	
	Fold	pvalue	Fold	pvalue	Fold	pvalue
Adipoq	0.91	0.583931	0.56	0.019147	0.59	0.029926
Bmp2	0.95	0.879185	0.6	0.002731	0.75	0.094598
Bmp4	0.81	0.425021	0.7	0.155405	0.61	0.074522
Bmp6	0.9	0.467973	0.68	0.001416	0.67	0.000393
Bmp7	1.24	0.148219	1.47	0.046466	1.58	0.01205
Ccl1	1.19	0.307056	1.14	0.33448	1.38	0.21628
Ccl11	0.59	0.017371	0.93	0.679976	1	0.867004
Ccl12	0.68	0.160694	1.45	0.048852	2.46	0.0235
Ccl17	0.94	0.450512	0.78	0.015756	1	0.913591
Ccl19	0.82	0.150721	0.86	0.333204	1.01	0.853258
Ccl2	1.02	0.988408	0.87	0.831197	1.47	0.061791
Ccl20	1.19	0.307056	1.14	0.33448	1.38	0.21628
Ccl22	0.89	0.461764	1.07	0.826792	0.9	0.513686
Ccl24	1.19	0.307056	1.14	0.33448	1.38	0.21628
Ccl3	0.95	0.636516	0.94	0.689664	1.28	0.141015
Ccl4	0.8	0.096693	1.2	0.234723	1.69	0.036897
Ccl5	1.17	0.301767	3.61	0.000556	4.17	0.000549
Ccl7	0.67	0.088605	0.87	0.543953	1.56	0.107773
Cd40lg	1.7	0.003046	1.73	0.011521	2.07	0.034439
Cd70	1.19	0.307056	1.14	0.33448	1.38	0.21628
Cntf	0.8	0.068851	0.8	0.048338	0.75	0.014198
Csf1	0.96	0.367275	0.82	0.08548	0.96	0.519101
Csf2	0.57	0.122848	1.03	0.880282	1.75	0.239301
Csf3	1.19	0.307056	1.2	0.186097	1.38	0.21628
Ctf1	0.95	0.832435	0.87	0.372823	0.98	0.969797
Cx3cl1	0.7	0.072427	0.76	0.073641	0.67	0.000696
Cxcl1	1.19	0.307056	1.14	0.33448	1.38	0.21628
Cxcl10	0.81	0.260633	1.37	0.203145	2.71	0.001359
Cxcl11	1.19	0.307056	1.14	0.33448	1.38	0.21628
Cxcl12	0.83	0.15215	0.67	0.009706	0.65	0.000679
Cxcl13	0.63	0.236418	1.02	0.79128	0.83	0.288971
Cxcl16	0.69	0.047622	1.24	0.219265	1.31	0.068874
Cxcl3	0.87	0.520423	0.94	0.763522	1.12	0.391076
Cxcl5	0.84	0.502853	1.49	0.279549	1.25	0.46346
Cxcl9	0.45	0.047223	1.46	0.317167	2.4	0.065739
Fasl	1.17	0.488506	1.87	0.023499	2.18	0.008328
Gpi1	1.1	0.22434	1	0.985763	1.02	0.796495
Hc	1.02	0.782101	0.93	0.51599	0.88	0.232293
Ifna2	0.68	0.163155	1.07	0.946324	1.54	0.286657
Ifng	1.13	0.394915	1.93	0.032028	2.28	0.003328
Il10	0.56	0.018905	1.32	0.240763	1.37	0.084617
Il11	1.19	0.307056	1.16	0.308112	1.38	0.21628
Il12a	0.87	0.219447	0.81	0.193625	0.76	0.062544
Il12b	1	0.93528	1.74	0.037167	1.68	0.015558
Il13	1.08	0.639405	1.13	0.451494	1.48	0.086936
Il15	1.15	0.115766	1.06	0.330977	1.25	0.003105
Il16	1.02	0.757687	0.96	0.686271	0.83	0.184723
Il17a	1.19	0.314393	1.14	0.34489	1.37	0.219546
Il17f	0.69	0.051111	0.98	0.956374	1.27	0.344276
Il18	0.74	0.009737	0.75	0.037951	0.81	0.059088
Il1a	0.74	0.074037	0.77	0.003672	0.78	0.02903
Il1b	0.81	0.037995	0.97	0.892349	1.02	0.768694
Il1rn	1.11	0.308385	0.99	0.891867	1.12	0.065731
Il2	1.19	0.307056	1.14	0.33448	1.38	0.21628
Il21	1.19	0.307686	1.77	0.093102	2.02	0.02555
Il22	1.19	0.307056	1.14	0.33448	1.38	0.21628
Il23a	0.9	0.561491	0.91	0.504591	1.04	0.734546
Il24	1.19	0.307056	1.14	0.33448	1.38	0.21628
Il27	1.16	0.288955	1.41	0.024535	1.33	0.051564
Il3	1.19	0.307056	1.14	0.33448	1.38	0.21628
Il4	1.88	0.120835	1.81	0.170613	2.33	0.048595
Il5	1.1	0.586481	1.12	0.49776	1.34	0.219948
Il6	1.37	0.228655	1.23	0.389211	1.45	0.130589
Il7	0.78	0.163456	0.61	0.074135	0.65	0.051514
Il9	1.16	0.417462	1.14	0.42832	1.66	0.204977
Lif	1.19	0.307056	1.14	0.33448	1.38	0.21628
Lta	1.01	0.88693	0.91	0.797704	0.91	0.71353
Ltb	1.19	0.083764	0.98	0.767313	1.07	0.461973
Mif	0.94	0.681914	0.91	0.366534	1.08	0.512092
Mstn	1.19	0.307056	1.14	0.33448	1.38	0.21628
Nodal	1.19	0.307056	1.29	0.151214	1.38	0.21628
Osm	0.72	0.010763	0.62	0.003199	0.63	0.003023
Pf4	0.89	0.763265	0.94	0.775812	1.06	0.625563
Ppbp	0.88	0.690144	0.93	0.614862	1.03	0.856684
Spp1	0.64	0.003612	0.69	0.002946	0.57	0.000723
Tgfb2	0.72	0.193137	0.76	0.075428	0.77	0.242564
Thpo	1.04	0.869218	1.26	0.21796	1.48	0.16488
Tnf	1.06	0.098276	1.09	0.131449	0.9	0.176216
Tnfrsf11b	1	0.983827	0.96	0.732539	1.26	0.311832
Tnfsf10	0.82	0.18516	1.14	0.219745	1.32	0.041119
Tnfsf11	0.76	0.04692	0.8	0.146692	0.92	0.699162
Tnfsf13b	1.06	0.296084	0.98	0.695247	1.08	0.195968
Vegfa	0.99	0.71329	0.99	0.917524	0.95	0.308337
Xcl1	0.99	0.774897	1.11	0.726891	1.38	0.137173

B

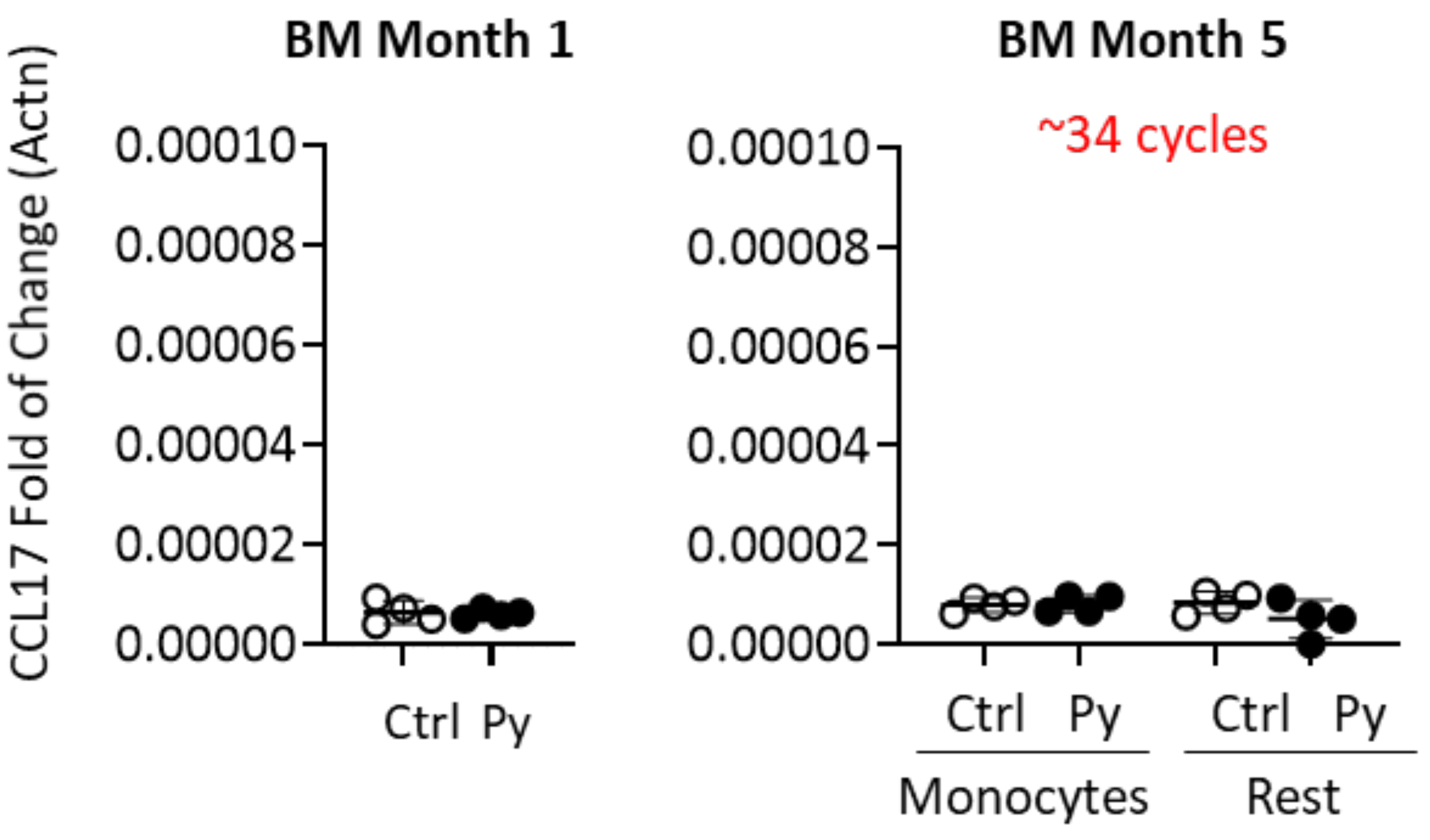

Figure S7. BM cytokine/chemokine array results in table format.
A) RNA expression values of cytokines and chemokines in BM cells at 2 months after infection with the indicated parasites using RT² Profiler™ PCR Array Mouse Cytokines & Chemokines (Qiagen). Genes with expression below the threshold (35 cycles are above) are shown in grey font. B-C) Real time PCR quantification of Ccl17 expression in BM cells from *Py*-infected or uninfected R2 mice one month after infection (B) or five months after infection (C). C) Monocytes were enriched using Monocyte Isolation Kit (BM, Miltenyi)

	R2 Py		R2 Pb Δpm4-Δbp2	
	Fold	p value	Fold	p value
Bcl6	1.49	0.524969	1.70	0.288560
Ccl11	0.71	0.645242	0.29	0.019389
Ccl5	3.04	0.049005	8.10	0.020813
Ccl7	0.44	0.356010	0.54	0.299365
Ccr10	0.58	0.183568	0.51	0.149066
Ccr2	1.55	0.335158	2.27	0.060139
Ccr3	2.21	0.050794	3.12	0.013026
Ccr4	1.11	0.645744	0.56	0.037248
Ccr5	1.91	0.056632	3.79	0.041459
Cd27	1.53	0.193090	1.92	0.210410
Cd28	1.52	0.432308	2.81	0.257207
Cd4	1.64	0.063952	2.05	0.074260
Cd40	0.75	0.324289	1.18	0.623987
Cd40lg	1.19	0.320267	1.14	0.365622
Cd80	1.33	0.433284	2.84	0.043794
Cd86	1.29	0.184852	1.58	0.042877
Cebpb	0.58	0.228714	1.71	0.034164
Crebbp	1.29	0.680584	1.47	0.453102
Csf2	1.28	0.639632	0.77	0.336167
Ctla4	2.19	0.159466	6.64	0.084597
Cxcr3	1.89	0.066024	2.21	0.051342
Fasl	2.24	0.130097	5.56	0.047109
Gata3	1.42	0.582709	0.98	0.796335
Gfi1	1.04	0.951370	2.01	0.096929
Icos	1.90	0.111798	2.14	0.207722
Ifng	2.06	0.137854	6.08	0.056425
Il10	1.57	0.332954	5.11	0.026360
Il12b	0.88	0.591053	1.89	0.065585
Il12rb2	1.02	0.956960	0.88	0.389020
Il13	0.92	0.728726	0.82	0.185383
Il13ra1	0.80	0.516540	1.22	0.334328
Il15	0.77	0.484117	0.70	0.253036
Il18	1.22	0.432814	0.93	0.797312
Il18bp	1.12	0.922447	1.70	0.144671
Il18r1	0.95	0.720157	1.02	0.748485
Il1r1	0.81	0.462503	0.94	0.728300
Il1rl1	0.88	0.947371	0.59	0.058803
Il2	1.11	0.695218	0.73	0.176346
Il25	0.92	0.728726	0.82	0.185383
Il27	0.85	0.529872	2.12	0.059286
Il27ra	1.16	0.681731	1.24	0.555398
Il2ra	1.00	0.986826	0.81	0.108376
Il3	0.92	0.728726	0.82	0.185383
Il4	0.85	0.964847	0.38	0.046051
Il4ra	0.98	0.798001	0.97	0.844638
Il5	0.88	0.882544	0.35	0.011067
Il6	0.87	0.386283	0.77	0.035181
Il7	0.88	0.557218	0.62	0.044194
Il7r	1.25	0.712517	0.82	0.571091
Il9	1.14	0.798495	0.33	0.105757
Irf1	1.07	0.579975	1.96	0.038387
Irf4	1.39	0.624983	2.38	0.074714
Jak1	1.06	0.726671	1.37	0.274116
Jak2	1.23	0.201145	1.51	0.005130
Jak3	1.54	0.282927	2.95	0.030977
Junb	1.43	0.625761	3.11	0.058525
Lta	1.05	0.957151	0.75	0.443141
Maf	1.02	0.822070	1.02	0.774419
Mapk8	1.03	0.882776	1.05	0.801737
Mapk9	1.00	0.980426	1.25	0.341607
Nfatc1	1.48	0.296282	1.80	0.148038
Nfatc2	1.37	0.250249	2.53	0.147289
Nfkb1	1.16	0.548933	1.59	0.089248
Pcgf2	0.88	0.193827	1.23	0.352785
Ptprc	1.19	0.453644	1.78	0.167744
Sftpd	1.24	0.405453	0.80	0.116262
Socs1	0.92	0.547550	1.37	0.291795
Socs3	0.75	0.250709	1.80	0.110799
Socs5	1.04	0.976426	1.02	0.943811
Spp1	0.44	0.217475	0.28	0.151336
Stat1	1.35	0.003738	2.25	0.003973
Stat4	1.47	0.434911	1.99	0.214450
Stat6	1.09	0.966135	1.37	0.444624
Tbx21	1.96	0.179606	4.08	0.168946
Tgfb3	0.80	0.426091	0.50	0.076330
Tlr4	1.15	0.044947	1.12	0.514235
Tlr6	1.11	0.828225	1.20	0.607685
Tnf	0.80	0.424710	1.56	0.218248
Tnfrsf4	0.92	0.728726	0.82	0.185383
Tnfrsf8	0.90	0.547119	0.96	0.906705
Tnfsf4	0.99	0.973762	1.96	0.198390
Tyk2	1.37	0.046083	1.69	0.119301
Vegfa	0.57	0.006610	0.76	0.035776
Yy1	1.21	0.171900	1.54	0.050213
Actb	1.12	0.663760	1.32	0.196097
B2m	1.40	0.516574	1.53	0.327995
Gapdh	1.22	0.745903	1.12	0.999340
Gusb	0.94	0.241525	1.09	0.330188
Hsp90ab1	1.00	0.923036	1.08	0.748130

Figure S8. Analysis of CD8$^+$ cells from BM: cytokine/chemokine array results in table format. RNA expression values of cytokines and chemokines in CD8$^+$ cells purified from BM cells at 6 weeks after infection with the parasite indicated above the graph using RT² Profiler™ PCR Array Mouse Th1/Th2 Cytokines & Chemokines (Qiagen). Genes with expression below the threshold (35 cycles are above) are shown in grey font.
